# Editorial: Human Ultra-Endurance Exercise

**DOI:** 10.3389/fphys.2020.00664

**Published:** 2020-06-25

**Authors:** Luca P. Ardigò, Carlo Capelli, Grégoire P. Millet

**Affiliations:** ^1^School of Exercise and Sport Science, Department of Neurosciences, Biomedicine and Movement Sciences, University of Verona, Verona, Italy; ^2^Institute of Sport Sciences, University of Lausanne, Lausanne, Switzerland

**Keywords:** mountain ultra-running bioenergetics, human performance limits, ultra-endurance exercise negative effects, nutrition countermeasures, running

Human Ultra-Endurance Exercise (i.e., any walking, running, swimming, kayaking, and/or cycling competition longer than 6 h) is a relevant topic both for basic human exercise psycho/physiology and for prescriptive aims in specific and diffused diseases (Scheer et al., 2020). New findings may prompt research focused on untrained or even pathological subjects in order to tailor extreme exercise for fitness and health purposes. This Research Topic includes six articles published belonging to three research areas: (1) mountain ultra-running bioenergetics, (2) human performance limits, and (3) ultra-endurance exercise negative effects and some nutrition countermeasures.

## Mountain Ultra-Running Bioenergetics

Vernillo, Savoldelli et al. assessed kinematics and metabolic variables [heart rate (HR), oxygen consumption (V°O_2_), and linear metabolic cost (C)] in 19 male ultra-marathon runners during uphill walking and running on treadmill post-to-pre the world's hardest mountain ultra-marathon (MUM). After MUM, kinematics variables did not change significantly. Differently, both HR and C decreased during both walking and running with V°O_2_ decreasing only during walking. Authors hypothesized that MUM prolonged and repeated walking and running caused a “generic” locomotion efficiency (eff) improvement reflected by the C decrease, i.e., a beneficial effect on running performance.

Vernillo, Millet et al. also reviewed the existing scientific literature on ultra-marathon's effect on metabolic cost, expressed either in metabolic (C-V°O_2_ [ml O_2_·kg^−1^·m^−1^]) or generic (C [J·kg^−1^·m^−1^]) energy unit. Some discrepancies were found since C is reported to increase or not after an ultra-marathon. Such differences may be due to methodological concerns. To address them, authors suggested standardizing running assessment over the many different ultra-marathon formats and environmental conditions, considering both sample's and different individuals' post-to-pre C changes, providing individuals with proper treadmill running familiarization, and involving as well a control group.

Savoldelli et al. ecologically investigated V°O_2_, HR, and GPS position of an experienced 50-year male ultra-marathon runner over six ascents (once *per* day) during a MUM. Data were used to calculated absolute and relative V°O_2_ and HR (compared with previously laboratory-assessed peak V°O_2_ and HR [V°O_2peak_ and HR_peak_]), C and vertical metabolic cost (C_vert_), and eff. Both metabolic (V°O_2_, HR, V°O_2peak_, and HR_peak_) and biomechanical (C, C_vert_, and eff) variables did not increase “consistently” over MUM.

## Human Performance Limits

Marck et al. questioned the current “limits” of *Homo sapiens* in terms of physical performances, lifespan and body height. Through historical analyses, they found out that humans have already approached their upper biological limits. Even acknowledging that some further improvement could be still achieved in terms of sport records, thanks to rules changes and/or technology progresses, authors state that reasonable mankind's challenge should now be to allow most people to reach the highest possible values of physical performances, lifespan, and body height.

## Ultra-Endurance Exercise Negative Effects and Some Nutrition Countermeasures

Zanchi et al. assessed MUM effect on brain water diffusivity—linked to cerebral *edema* risk—and blood composition in 19 finishers by using magnetic resonance and analyzing blood samples. Overall, even 2 days after arrival brain did not recover to its pre-MUM state confirming the destabilizing nature of mountain ultra-endurance exercise. This was an indirect consequence of the inflammation-driven increase in extracellular water in the body.

Qin et al. investigated the effect of alpha-lactalbumin and whey protein on muscle damage and pain, and mood during a 4-h recovery in 12 endurance male runners following prolonged strenuous exercise (i.e., 90 min at *maximum* V°O_2_). Carbohydrate+alpha-lactalbumin (CA) or +whey protein (CW) were administrated during the recovery first 2 h. As a whole, CA ingestion induced decreases in muscle pain, fatigue feeling, and cortisol response during recovery after high-intensity long-lasting running.

## Author Contributions

All authors listed have made a substantial, direct and intellectual contribution to the work, and approved it for publication.

## Conflict of Interest

The authors declare that the research was conducted in the absence of any commercial or financial relationships that could be construed as a potential conflict of interest.
